# Dynamic development of starch granules and the regulation of starch biosynthesis in *Brachypodium distachyon*: comparison with common wheat and *Aegilops peregrina*

**DOI:** 10.1186/s12870-014-0198-2

**Published:** 2014-08-06

**Authors:** Guanxing Chen, Jiantang Zhu, Jianwen Zhou, Saminathan Subburaj, Ming Zhang, Caixia Han, Pengchao Hao, Xiaohui Li, Yueming Yan

**Affiliations:** College of Life Science, Capital Normal University, 100048 Beijing, China

**Keywords:** *Brachypodium* Bd21, B-granules, Starch biosynthesis, Expression profiling, GBSSI, Phosphorylation

## Abstract

**Background:**

Thorough understanding of seed starch biosynthesis and accumulation mechanisms is of great importance for agriculture and crop improvement strategies. We conducted the first comprehensive study of the dynamic development of starch granules and the regulation of starch biosynthesis in *Brachypodium distachyon* and compared the findings with those reported for common wheat (Chinese Spring, CS) and *Aegilops peregrina*.

**Results:**

Only B-granules were identified in *Brachypodium* Bd21, and the shape variation and development of starch granules were similar in the B-granules of CS and Bd21. Phylogenetic analysis showed that most of the Bd21 starch synthesis-related genes were more similar to those in wheat than in rice. Early expression of key genes in Bd21 starch biosynthesis mediate starch synthesis in the pericarp; intermediate-stage expression increases the number and size of starch granules. In contrast, these enzymes in CS and *Ae. peregrina* were mostly expressed at intermediate stages, driving production of new B-granules and increasing the granule size, respectively. Immunogold labeling showed that granule-bound starch synthase (GBSSI; related to amylose synthesis) was mainly present in starch granules: at lower levels in the B-granules of Bd21 than in CS. Furthermore, GBSSI was phosphorylated at threonine 183 and tyrosine 185 in the starch synthase catalytic domain in CS and *Ae. peregrina*, but neither site was phosphorylated in Bd21, suggesting GBSSI phosphorylation could improve amylose biosynthesis.

**Conclusions:**

Bd21 contains only B-granules, and the expression of key genes in the three studied genera is consistent with the dynamic development of starch granules. GBSSI is present in greater amounts in the B-granules of CS than in Bd21; two phosphorylation sites (Thr183 and Tyr185) were found in *Triticum* and *Aegilops*; these sites were not phosphorylated in Bd21. GBSSI phosphorylation may reflect its importance in amylose synthesis.

**Electronic supplementary material:**

The online version of this article (doi:10.1186/s12870-014-0198-2) contains supplementary material, which is available to authorized users.

## Background

Starch is the major storage carbohydrate in the seeds of cereal crops. Starch comprises approximately 90% and 65–75% of the dry weight of rice and wheat, respectively [1]. Starch consists of the glucose polymers amylose and amylopectin. Amylose is a relatively linear molecule consisting of (1–4)-linked units of D-glucopyranosyl, whereas amylopectin mainly consists of long chains of (1–4)-linked D-glucopyranosyl units with occasional branching (1–6) linkages that yield tandem linked clusters (~9–10 nm long each) [2]. In the current model of the multiple-cluster structure of amylopectin, A-chains are linked to other chains at their reducing ends, whereas B-chains carry 1 or more chains belonging to a cluster. B1-chains are present within single clusters, whereas B2- and B3-chains are long chains interconnecting many clusters. The only chain that contains a reducing terminal in an amylopectin molecule is called a C-chain [3]. Amylopectins from different species exhibit different chain length distributions with periodic occurrence of varying degrees of polymerization (DP). These chains are grouped into four fractions with DP in intervals 6–12 (A-chain), 13–24 (B1-chain), 25–36 (B2-chain), and >37 (B3- or more advanced chains) [4].

The endosperm of mature wheat (*Triticum aestivum* L.) contains three types of starch granules: A, B, and C. A-granules, from 10 to 50 μm in diameter, constitute up to 70% of the volume and 10% of the total number of starch granules [5,6]. In contrast, B-granules, 5–9 μm in diameter, constitute approximately 30% of the volume and 90% of the total number of granules. Recent evidence indicates the presence of C-granules with a diameter less than 5 μm; their small size makes them difficult to isolate and quantify, which commonly leads to them being classified with B-granules [7,8]. In wheat, B-granules negatively affect flour processing and bread quality [9], but positively affect pasta production [10]. This is thought to be due, at least in part, to the swelling capacity of B-granules: they bind more water than A-granules do [11]. The A- and B-granules in the *Triticeae* endosperm are separated in time and space. A-granules are formed approximately 4–14 days post-anthesis (DPA) when the endosperm is still actively dividing [12,13]. B-granules appear approximately 10–16 DPA, whereas the small C-granules first appear ~21 DPA [6,7]. The genetic basis of the multimodal size distribution of starch in wheat and barley is of great interest because the physiochemical properties of each type of granule vary and contribute to the food and industrial end uses of *Triticeae* starch [14–16].

Amylose synthesis is controlled by granule-bound starch synthase (GBSSI) [17]. Amylopectins are synthesized by concerted reactions catalyzed by four enzyme classes: ADP-glucose pyrophosphorylase (AGPase), starch synthase (SS), starch-branching enzyme (SBE), and starch-debranching enzyme (DBE). AGPase catalyzes the first reaction in starch synthesis, producing the activated glucosyl donor ADP-glucose. Starch synthases catalyze transfer of glucose units from ADP-glucose onto the non-reducing end of a glucan chain to synthesize water-insoluble glucan polymers [18]. In cereal species, starch synthases are subdivided into granule-bound starch synthase (GBSS) and SS, responsible for amylopectin synthesis. GBSS is the only SS found exclusively within the starch granule and responsible for amylose synthesis [17]. The SS group consists of four isoforms designated SS-I, SS-II, SS-III, and SS-IV, which are localized predominantly at the granule surface [19]. Genetic analyses of *Arabidopsis* and rice suggest SS-I is required for the elongation of short A-chains within amylopectin [20,21]. The function of SS-II is the elongation amylopectin chains of DP 6–10 to produce intermediate-length chains of DP 12–25 [22]. Analysis of SS-III mutants suggests this enzyme class catalyzes the synthesis of long amylopectin chains, DP 25–35, or greater [23–25]. Although little is known about the role of SS-IV in starch synthesis, recent research in *Arabidopsis* showed that it may function to control granule number [26]. Starch-branching enzyme isoforms SBEI and SBEII generate *α* (1, 6) linkages that form the branched structure of amylopectin. SBEI plays an important but not exclusive role in the synthesis of B1-, B2-, and B3-chains. The *SBEII-a* and *SBEII-b* genes also perform a distinct function in the formation of A-chains [27–29]. Two groups of DBEs exist in plants: isoamylase type and pullulanase type (also known as limit dextrinases), which efficiently hydrolyze (debranch) α-(1–6)-linkages in amylopectin and pullulan (a fungal polymer of malto-triose residues), respectively, and belong to the α-amylase superfamily. One of the starch debranching enzymes, isoamylase (ISAI), is an essential player in the formation of crystalline amylopectin [18]. Pullulanase can supplement the function of isoamylase to some extent.

The genome sequence of *Brachypodium distachyon* L. was completed in 2010; analysis suggests *Brachypodium* is much more closely related to wheat and barley than to rice, sorghum, or maize [30,31]. In-depth studies of starch are necessary and significant because starch is a major storage carbohydrate in the seeds of cereal crops. Until now, considerable research has focused on various characteristics of *Brachypodium*, but the properties and development of starch granules remains poorly studied. We performed a comprehensive survey of the dynamic development of starch granules and regulation of starch synthesis in *Brachypodium* through comparative analysis with *Triticum* and *Aegilops*. We also studied the phosphorylation status of GBSSI, which controls amylase synthesis. Our results provide new insights into the molecular mechanisms of starch granule development and starch biosynthesis.

## Results

### Development of grains and starch granules in *Brachypodium*

The morphological features and dynamic changes in developing grains during 13 stages after flowering in Bd21, Chinese Spring (CS), and *Ae. peregrina* are shown in Additional file 1. In all three genera, grain size and weight gradually increased from flowering to maturity, but some developmental differences were apparent. The grains were rapidly elongated from 2 to 8 DPA in Bd21 and from 2 to 12 DPA in CS and *Ae. peregrina*; at subsequent developmental stages, grain length increased slightly, while grain width and weight gradually increased until maturity (Additional file 1A). Bd21 grain weight increased slightly throughout development, but increased rapidly from 2 to 20 DPA in CS and *Ae. peregrina*. At 30 DPA, the grain weight reached the highest value (Additional file 1B).

**Figure 1:**
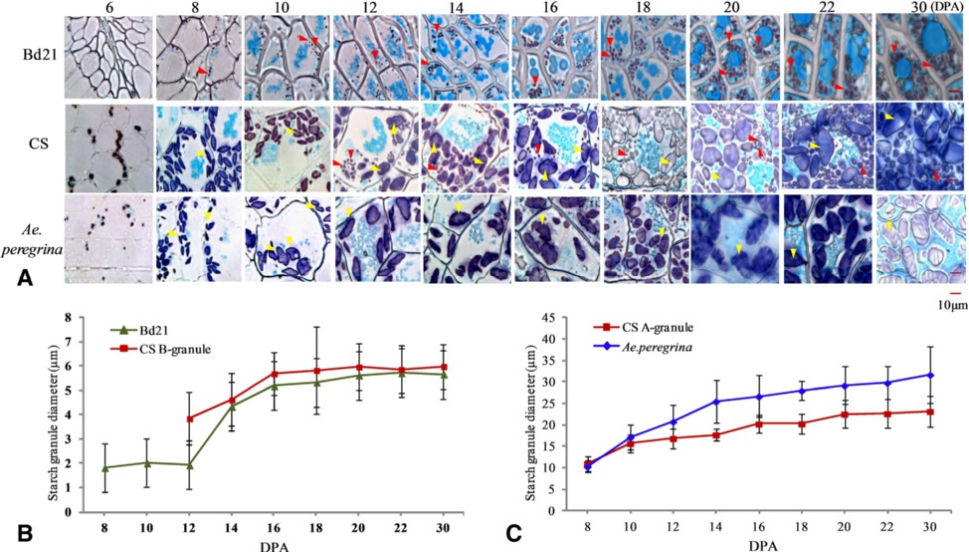
**Observation and statistics of starch granules diameter during development of seeds. A**, Bright-field images of grain cross-sections stained with Fast Green and iodine allowing for the visualization of both intracellular proteins (green) and starch (blue-purple). The yellow arrows show A-granule starch, and the red arrows point to B-granule starch. **B**, Diameter of starch granules during development of seeds: comparison between A-granule starch granules of Chinese Spring (CS; common wheat) and those of *Aegilops peregrina*. DPA: days post-anthesis. **C**, Diameter of starch granules during development of seeds: comparison between B-granule starch granules of CS and of *Brachypodium distachyon* Bd21.

The dynamic accumulation patterns of starch granules in the grain endosperm and pericarp during grain development were examined by light microcopy and SEM. In this study, plenty of starch appeared in the pericarp at the beginning of the seed formation. As shown in Additional file 2, there was a thick pericarp layer with abundance of starch at 4 DPA that persisted through 12 DPA. Colored starch grains were observed throughout the stages of grain development (Figure 1A). In Bd21, the starch granules appeared ~8 DPA; their diameter remained less than 10 μm throughout growth and were thus classified as B-granules (Figure 1B). The starch granules in CS grew rapidly from 6 to 8 DPA but remained less than 10 μm in diameter; growth slowed from 8 to 12 DPA and yielded granules of diameter greater than 10 μm; these were classified as A-granules.

**Figure 2:**
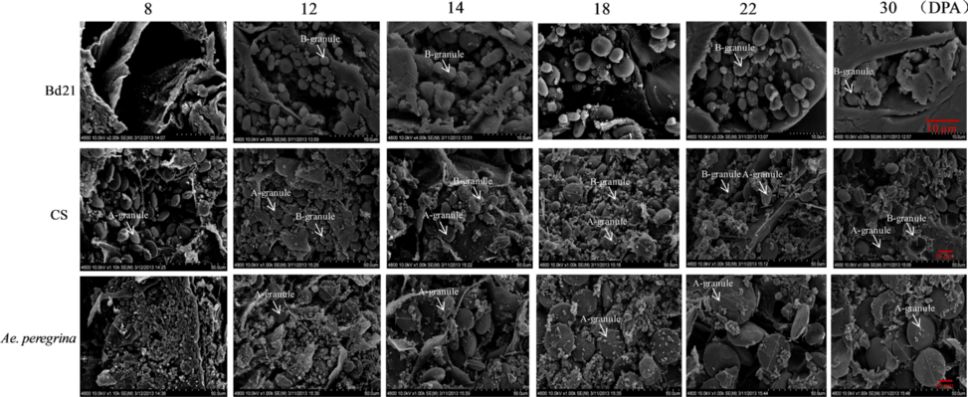
**SEM images of grain cross-sections during grain development.**

The B-granule, whose diameter was less than 10 μm, appeared at 12 DPA. These 2 kinds of starch granules gradually increased during the subsequent period with the average diameter of A-granules stabilized at 20–30 μm and the diameter of B-granules at approximately 4–6 μm. The average granule diameter reached 10 μm by 10 or 12 DPA in *Ae. peregrina*; these were classified as A-granules (Figure 1C). SEM of the variation in starch shape during grain development confirmed these results (Figure 2).

**Figure 3:**
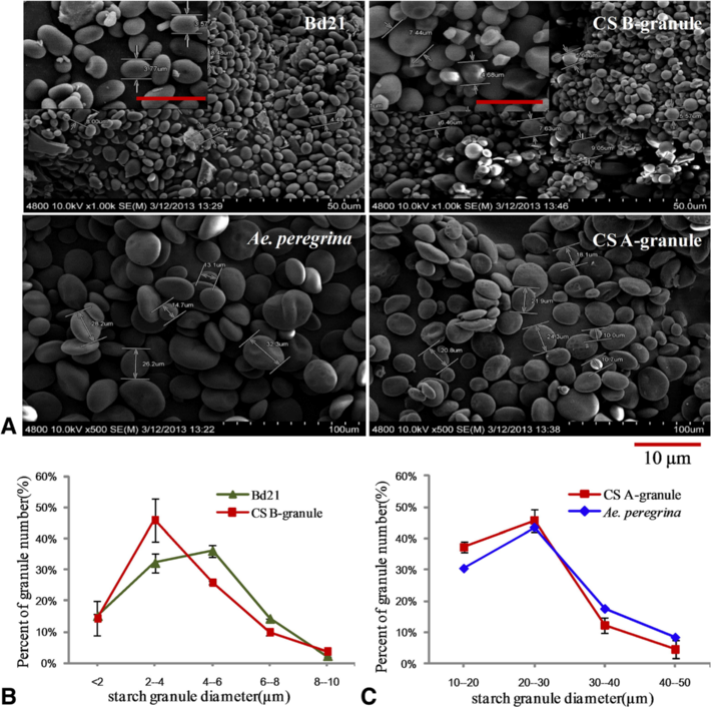
**The distribution of diameters of starch granules in mature seeds. A**, SEM of purified granules of *Brachypodium distachyon* Bd21 and *Aegilops peregrina* and of A-granule and B-granule starch granules of Chinese Spring (CS; common wheat). The scale bar is 10 μm*.* **B**, The distribution of diameters of starch granules among A-granule starch granules of CS and *Ae. peregrina*. **C**, The distribution of diameters of starch granules among B-granule starch granules of Bd21 and CS.

In order to confirm that there are only A-granules in *Ae. peregrina* and B-granules in Bd21, we purified all the granules from Bd21 and *Ae. peregrina*, and A-granules and B-granules from CS (Figure 3A). Statistical analysis showed that granule diameter in Bd21 ranged from 4–6 μm, similar to the B-granules of CS (Figure 3B), whereas the diameter of starch granules in *Ae. peregrina* ranged from 20–30 μm, similar to the A-granules of CS (Figure 3C).

### Chromosomal localization, domain conservation, and phylogenetic analysis of starch synthesis-related genes in *Brachypodium*

To identify the key genes regulating starch biosynthesis, the consensus amino acid sequences previously annotated in rice, wheat, and maize were used to perform a BLAST search against the whole *Brachypodium* genome database (http://www.brachypodium.org/). Twenty-four nonredundant enzymes related to starch synthesis were identified. Their distribution on the five *Brachypodium* chromosomes and their domain structures are shown in Additional files 3 and 4. The starch synthesis-related enzymes were distributed among five chromosomal regions, seven of which (*AGPII-b, SBEI, SBEIII, SSII-a, SSI, GBSSI*, and *AGPL IV*) were located on chromosome 1 from 0 to 74.8 Mb, four genes (AGPLI*, SSIV-b, ISAIII, and *GBSSII*) on chromosome 2 from 0 to 59.3 Mb, six genes (*SSIII-a, SSII-c, ISAI, SBEII-a, AGPLIII*, and *SSII-b*) on chromosome 3 from 0 to 59.8 Mb, three genes (*SSVI-b, AGPSI*, and *ISA II* ) on chromosome 4 from 0 to 48.6 Mb, and four genes (*PUL, SBEII-b, AGPSII-a*, and *SSIII-b*) on chromosome 5 from 0 to 28.1 Mb (Additional file 3).

As shown in Additional file 4, starch synthases including GBSSI, GBSSII, SSI, SSII-a, SSII-b, SSII-c, SSIII-a, SSIII-b, and SSIV-b, are mainly composed of two structural domains: the starch synthase catalytic domain and the glycosyl transferase domain. SSIII-a and SSIII-b have a redundant carbohydrate-binding domain at the N terminus. SBEs and DBEs (except PUL) shared greater similarity, and all had the carbohydrate-binding module and an α-amylase catalytic domain, but the SBEs contained one more α-amylase C-terminal all-β domain at the C terminus. PUL is comprised of a carbohydrate-binding domain, α-amylase catalytic domain, and a domain with an unknown function. ADP-glucose pyrophosphorylase small subunit (AGPS) had only one nucleotidyl transferase domain, whereas the ADP-glucose pyrophosphorylase large subunit (AGPS) contained a ribosomal protein L11 N-terminal domain and a ribosomal protein L11 RNA-binding domain (Additional file 4).

In order to understand the relationships among the 70 genes associated with starch synthesis in *Brachypodium*, rice, wheat, and maize, we constructed a phylogenetic tree (Additional file 5a). The genes were clearly separated into two groups: Group I included SSs and SBEs, whereas Group II consisted of DBEs and AGPases. Some key genes for starch synthesis were selected to construct different phylogenetic trees, including *GBSSI*, *SSI*, *SBEI*, *SBEII-a*, *ISAI*, *PUL*, and *AGPL* (Additional file 5b–g). Although the genes related to starch synthesis from *Brachypodium*, rice, wheat, and maize showed high similarity, most genes from *Brachypodium* were closer to those of wheat than rice and maize.

### Dynamic expression profiles of starch synthesis-related genes during grain development

**Figure 4:**
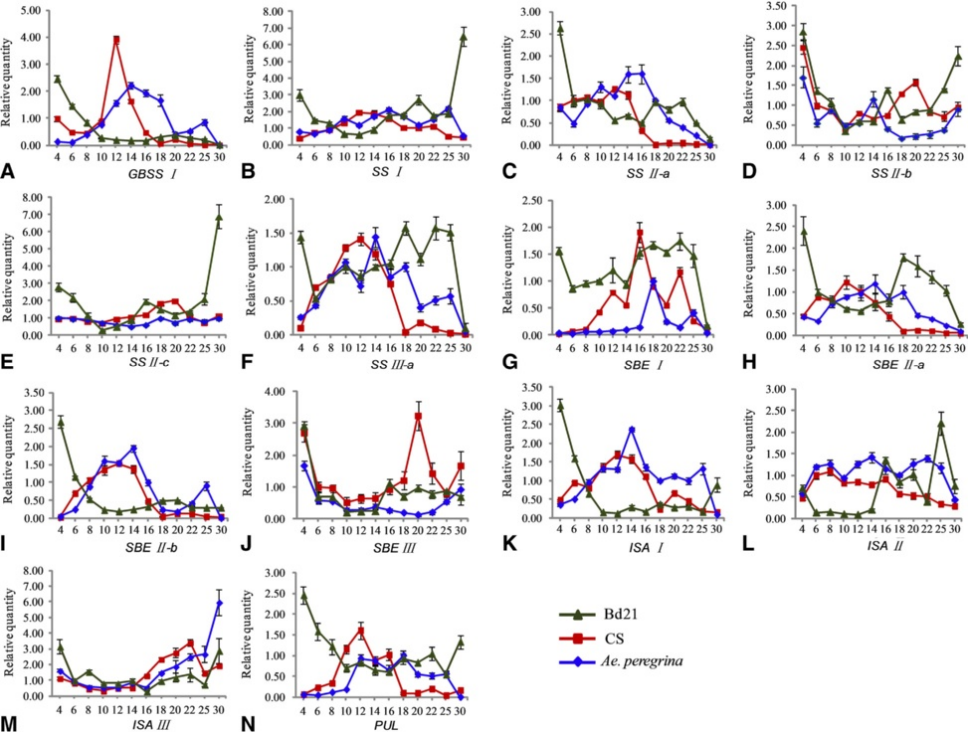
**qRT-PCR analysis of genes related to starch synthesis in developing seeds.** Trangle, *Brachypodium distachyon* Bd21; square, CS (Chinese Spring); rhombus, *Aegilops peregrina*.

The dynamic expression profiles of 14 main starch synthesis-related genes during 12 grain developmental stages in *Brachypodium* Bd21 as well as common wheat (CS) and *Ae. peregrina* were analyzed by qRT-PCR (Figure 4A-N) and melt curve analysis. Although the genes showed some similarities, their expression patterns were distinct during grain development in each of the studied genera. We observed six expression patterns: Type I (down-up), Type II (up-down), Type III (down-up-down), Type IV (up-down-up-down), Type V (down-up-down-up), and Type VI (up-down-up-down-up-down) (Table 1).

**Table 1:** **Expression pattern of the 14 genes in** ***Brachypodium distachyon*** **Bd21, Chinese Spring (CS; common wheat), and** ***Aegilops peregrina***

**Pattern**	**Bd21**	**CS**	***Ae. peregrina***
Type I (Down-up)	*GBSSI, SSI, SBEIII, ISAI, ISAII, ISAIII, PUL*	*ISAIII*	*ISAIII*
Type II (Up-down)		*GBSSI, SSI, SSII-a, SSIII-a, SBEI, SBEII-a, SBEII-b, ISAI, ISAII, PUL*	*GBSSI, SSI, SSII-a, SSIII-a, SBEI, SBEII-a, ISAI, PUL*
Type III (Down-up-down)	*SSII-a, SSIII-a, SBEI, SBEII-a, SBEII-b*		
Type IV (Up-down-up-down)			*SBEII-2b*
Type V (Down-up-down-up)	*SSII-b, SSII-c*	*SSII-b, SSII-c, SBEIII*	*SSII-b, SSII-c, SBEIII*
Type VI (Up-down-up-down- up-down)			*ISAII*

Starch is composed of glucose polymers amylose and amylopectin. *GBSSI*, controlling amylose synthesis, displayed the down-up expression pattern (Type I) in Bd21 and exhibited higher early expression (4–8 DPA) and weaker expression at later stages (10–30 DPA). In contrast, *GBSSI* exhibited an up-down expression trend (Type II) and was mainly expressed at the intermediate stages of growth in wheat and *Ae. peregrina* (Figure 4A). Amylopectin synthesis is mainly controlled by SSs, SBEs, and SDEs. Two expression patterns (Type I and Type III) were exhibited in Bd21: the starch synthase (*SSII-a* and *SSIII-a*) and starch branching enzyme (*SBEI, SBEII-a* and *SBEII-b*) mainly exhibited a Type III expression pattern, whereas starch branching enzymes *ISAI, ISAII, ISAIII,* and *PUL* displayed a Type I expression pattern (Table 1). For example, *SSII-a* and *SSIII-a* showed a down-up-down expression trend (Type III) in Bd21, and was strongly expressed at 4 DPA and 18–25 DPA, and then moderately expressed during grain filling (8–16 DPA), but minimally expressed at 30 DPA (Figure 4C and 4F). *ISA I* and *PUL* exhibited a down-up pattern (Type I) in Bd21: expression was very strong at 4 DPA, decreased rapidly at 10 DPA, stabilized at the later stages, and then increased at 30 DPA (Figure 4K and 4N). However, Type II was the main expression pattern observed in wheat and in *Ae. peregrina.* For instance, *ISA I* and *PUL* showed an up-down expression trend and were mainly expressed at the intermediate stages in wheat and at intermediate late stages in *Ae. peregrina* (Figure 4K and 4N). *SS-I* displayed a Type I expression pattern in Bd21: down-regulation from 4 to 12 DPA and up-regulation from 12 to 30 DPA. In contrast, it exhibited an up-down pattern from 4 to 30 DPA and was expressed at lower levels in wheat and *Ae. peregrina* (Figure 4B). *SSII-b* and *SSII-c* exhibited the Type V expression trend (up-down-up-down) in all three genera (Figure 4D and 4E).

### Western blot analysis and immunolocation of GBSSI

**Figure 5:**
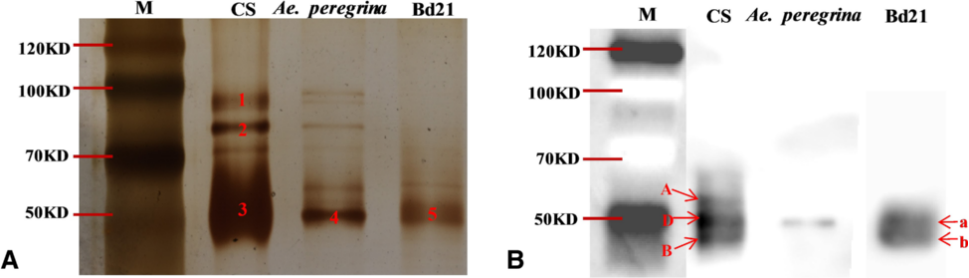
**Isolation and identification of amylase in CS,** ***Ae. peregrina*** **, and Bd21. A**, SDS-PAGE of amylase extracted from *Brachypodium distachyon* Bd21, Chinese Spring (CS; common wheat), and *Aegilops peregrina*. **B**, Western blot analysis of the granule-bound starch synthase (GBSSI) protein in CS, *Ae. peregrina*, and Bd21.

GBSSI is a key enzyme in amylase synthesis, and therefore it affects the physicochemical properties of flour and its end-products. Starch granule-binding proteins were extracted and fractionated by SDS-PAGE and silver-stained (Figure 5A). The isolated GBSSI was confirmed using matrix-assisted laser desorption ionization time-of-flight mass spectrometry (MALDI-TOF/TOF MS) (Additional file 6). The monoclonal antibodies against GBSSI (i.e., against its peptide) demonstrated high specificity to GBSSI (Figure 5B). The results showed three kinds of GBSSI in CS, corresponding to A, D, and B types [32] (Figure 5B). One and two protein bands were observed in *Ae. peregrina* and Bd21, respectively.

**Figure 6:**
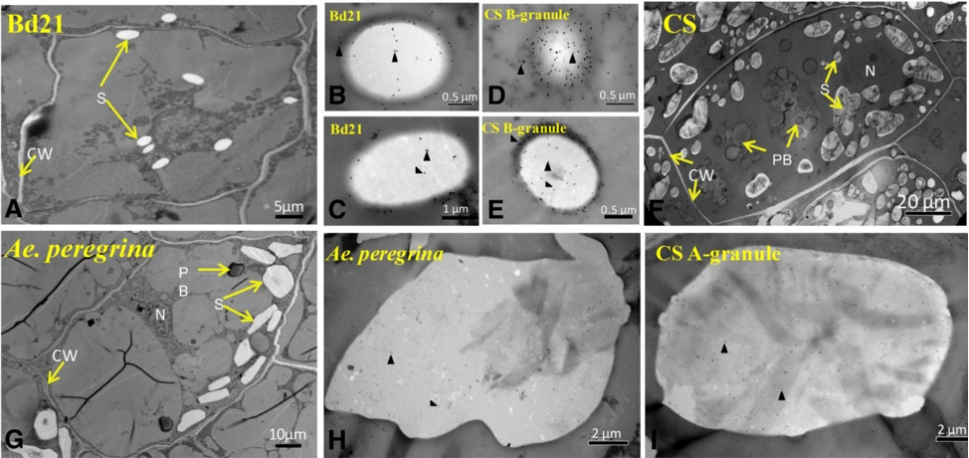
**Immunolocalization of GBSSI in immature seeds (12 days post-anthesis [DPA]). A**, **F** and **G**, Morphological observations. **B-E**, Immunocytochemical observation of B-granules. **H-I**, Immunocytochemical observation of A-granules. S, starch granules; PB, protein body; CW, cell wall; N, nucleus. Triangular arrowheads indicate gold particles.

Immunogold labeling was used to determine the subcellular localization and the amount of GBSSI in Bd21, CS, and *Ae. peregrina.* Ultrathin sections of 12-day-old immature seeds were processed as described in Methods. As shown in Figure 6, GBSSI was detected mainly in the starch granules of immature seeds. The amount of GBSSI in the B-granules of CS was greater than in Bd21, but the amount of GBSSI was similar in the A-granules of CS and *Ae. peregrina.*

### Phosphorylation of GBSSI in starch granules during grain development

**Figure 7:**
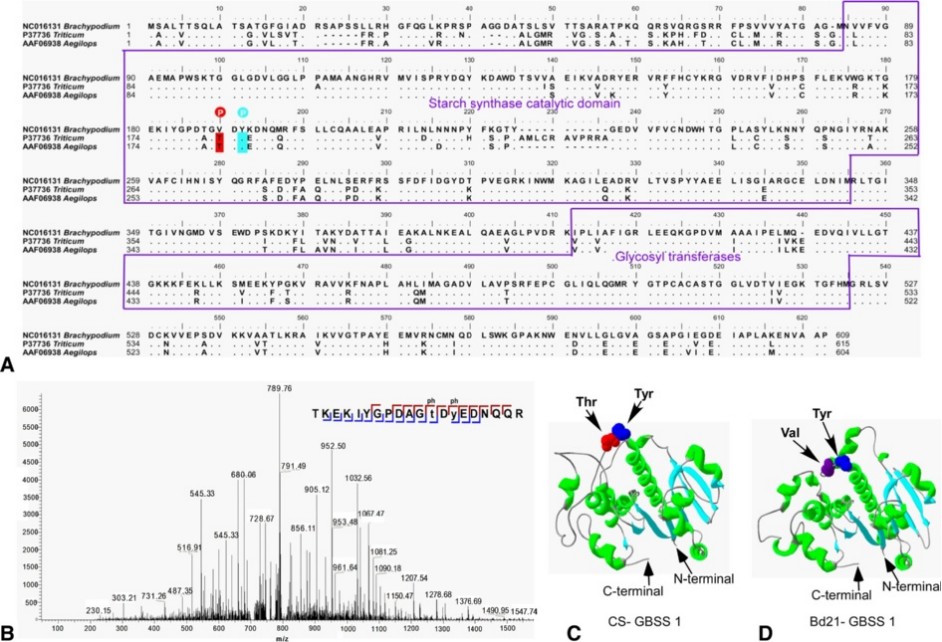
**Phosphorylation of GBSSI. A**, Amino acid sequence alignment of granule-bound starch synthases (GBSSI proteins). The phosphorylated residues are marked. **B**, The mass spectrometric spectrum of the phosphopeptide. **C**, 3D structure is shown for GBSSI of Chinese Spring (CS; common wheat) and *Aegilops peregrina*. **D**, 3D structure is shown for GBSSI of *Brachypodium distachyon* Bd21.

In this study, we detected two phosphorylated peptides: one at threonine 183 and one at tyrosine 185 in GBSSI of CS and *Ae. peregrina* (Additional file 7). The threonine and tyrosine residues were all located in the starch synthase catalytic domain (Figure 7A). However, no phosphorylation at this position was observed in Bd21. As shown in Figure 7A, the phosphorylated threonine in *Triticum* and *Aegilops* was replaced by valine in *Brachypodium*; this substitution may be responsible for the absence of phosphorylation in Bd21. The MS spectrum of a relevant phosphopeptide (Figure 7B) confirmed this result. The 3D structure of GBSSI (Figure 7C and 7D) was predicted using Phyre^2^ (http://swissmodel.expasy.org) and revealed the phosphorylated and unphosphorylated sites of CS and Bd21. The figure shows that in the 3D model, structurally relevant amino acids forming the starch synthase catalytic domain are well conserved. There are 12 α-helices and 11 β-strands in the starch synthase catalytic domains of CS and Bd21, and the phosphorylated amino acid was always located between the third and fourth helix.

## Discussion

### *Brachypodium* has only B-granules

In mature wheat (*Triticum aestivum* L.), the endosperm contains three types of starch granules: A-granules 10–50 μm in diameter and B-granules (including C-granules) less than 10 μm in diameter [8]. Previous studies confirmed that A-granules are formed at approximately 4–14 DPA and B-granules start to appear at approximately 10–16 DPA [6–8]. In this study, the starch granules in Bd21 appeared ~8 DPA, and their diameters were remained 4–6 μm until maturity. Thus, all starch granules in Bd21 are B-granules. In contrast, Guillon *et al*. [33] showed that Bd21 starch granules start to appear at ~17 DPA. This is a bit longer than our observation, probably because of differences in growth conditions. B-granules in CS appeared at ~12 DPA, and then, they grew slowly. Their diameter was mostly in the range of 4–6 μm. A-granules in CS showed rapid growth at early stages and reached 10 μm at 10 DPA, and the diameter was mostly stable at 20–30 μm. Starch granules in *Ae. peregrina* appeared early and reached a diameter up to 10 μm at 10 DPA; all starch granules in *Ae. peregrina* were A-granules, as reported previously [34]. Thus, CS had both A- and B-granules whereas *Ae. peregrina* and Bd21 contained only A-granules or B-granules.

*Brachypodium*, *Triticum*, and *Aegilops* are closely related, although the sizes of their starch granules differ. The varied composition of A- and B-granules as well as diverse A:B granule ratios in *Brachypodium*, *Triticum*, and *Aegilops* suggest some genes specifically control the formation of A-granules and B-granules [35]. In wheat, a quantitative trait locus (QTL) associated with granule size was found on chromosome 4B [36], and the QTLs affecting the A:B ratio of granules are located on chromosome 4DS [37]. In barley, a QTL affecting the shape of B-granules was identified on chromosome 4H [38]. A recent study showed that a major QTL controlling the content of B-granules is located approximately 40 cM on the short arm of chromosome 4S of *Aegilops* [34]. Those authors speculate that it is the tetraploidization event that leads to inactivation of the B-granule loci [34]. However, B-granules exist in all the diploid, tetraploid, and hexaploid lines of *Brachypodium*; thus, the polyploidization event may not be responsible for the lack of a B-granule site in *Brachypodium*. We speculate that the genes controlling A-granule loci may be silenced/deleted during evolution. A recent study showed that *Brachypodium* has a highly conserved seed storage protein Gli-2 as well as a Glu-1 and a Glu-3 locus just like in *Triticum* and the related species, but almost no protein is detected because of abundant premature stop codons [39–41]. Moreover, previous analysis of *Hardness-like* genes, the main determinants of the grain softness/hardness trait in wheat, showed that *Hardness-Brachy* genes in *Brachypodium* could have been deleted independently during evolution [42]. We also theorize that the genes controlling A-granules may have been independently deleted/silenced when *Brachypodium* and *Triticeae* diverged nearly 35 million years ago [43,44]. Finally, from the standpoint of morphology, because *Brachypodium* cells are much smaller than those of other cereals, the larger A-granules are too hard to support. The major QTL controlling the content of A-and B-granules has not been identified, and further research to map and identify the gene(s) responsible for A- or B-granule initiation remains to be done.

### Expression of starch synthesis-related genes and starch biosynthesis

**Figure 8:**
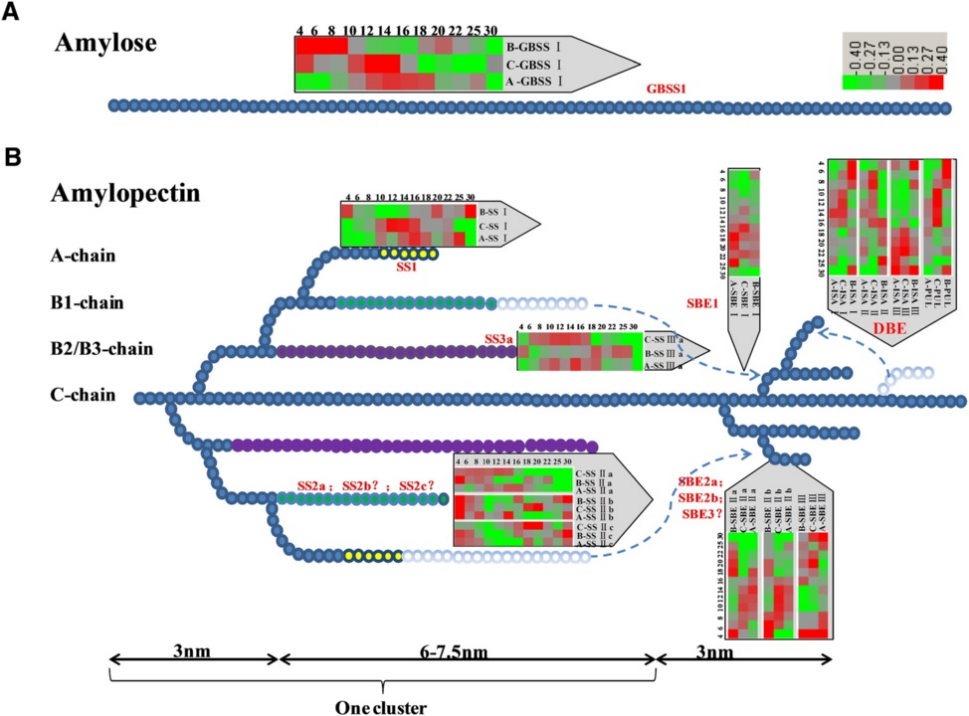
**Synthesis of (A) amylose and (B) an amylopectin cluster in the endosperm.** Starch synthase I (SSI): catalyzes the synthesis of elongated amylopectin chains with the degree of polymerization (DP) of approximately 6–7, to form chains of DP 8–12. SSII-a: catalyzes the synthesis of elongated amylopectin chains of DP 6–10 to DP 12–25. SSIII catalyzes the synthesis of long amylopectin chains of DP 25–35 or greater. Starch-branching enzyme I (SBEI) plays an important but not exclusive role in the synthesis of B1-, B2-, and B3 chains. SBEII-b performs a distinct function in the formation of A-chains. Debranching enzymes (DBEs) remove unnecessary or erroneous branches.

The high expression level of starch synthesis-related genes at very early stages in *Brachypodium* attracted our attention. Other studies have shown that in the outer layers of cereal grains, starch accumulates transiently at the beginning of grain development, where it contributes to carbon storage during the earlier phases [45]. Nakamura *et al*. [46] reported that there is a thick pericarp layer with abundance of starch at 5 DPA, which persists until 20 DPA in wheat. The starch growth prevails in the early pericarp (0–4 DAF), then degenerates from 6 DAF [47]. In our study, abundant starch appeared in the pericarp at the beginning of seed formation (Additional file 2). As in barley, almost all genes showed low expression at 4 DPA in CS and *Ae. peregrina*, even though there were four genes, including *SSIII-a, SBEI, SBEII-b, PUL*, whose expression was nearly undetectable [48]. On the other hand, all of the 14 genes displayed high expression in Bd21 at 4 DPA (Figure 8). The high level of expression of genes during the early stages in Bd21 may be responsible for the production and accumulation of starch in the pericarp.

Nevertheless, these genes showed a relatively different expression pattern in the endosperm of these 3 genera. The amount of starch in the developing Bd21 seeds increased steadily between 8 and 20 DAF; in particular, the amount of starch showed an obvious increase at 16 and 18 DPA. At the same time, most of the genes exhibited high expression at approximately 16–18 DPA (Figure 8). The expected expression of starch synthesis-related genes (especially *SSI*, *SSII-a*, *SSIII-a*, *SBEI*, *SBEII-a*, and *SBEII-b*) also appeared at 16 to 18 DPA, and may be responsible for the synthesis of B-granules and increase of the endosperm. In this study, genes controlling synthesis of B-chains were expressed earlier than the genes related to A-chains. As shown in Figures 1 and 8, *SSII-a* (controls synthesis of B1-chains), *SSIII-a* (controls synthesis of B2-chains) and *SBEI* (controls synthesis of B1/B2-chains or others) were expressed earlier than *SS-I* and *SBEIIa* and *SBEIIb* (control synthesis of A-chains). A-chains are linked to B-chains; therefore, B-chains should be synthesized early to provide support for A-chains. Although in CS, strong expression mostly appeared ~12 DPA, which was responsible for the synthesis of B-granules, it is unexpected that *SBEI* was expressed later than *SBEII-a* and *SBEII-b*. The same phenomenon was also observed in *Ae. peregrina*: *SBEI* was expressed later than *SBEII-a* and *SBEII-b* were*.* Since the sequence of SBEs in *Brachypodium*, wheat, rice and maize showed a high similarity and were classified into the same cluster, respectively (Additional file 5). We can hypothesize that SBEs in CS and *Ae. peregrina* have the functions that are opposite to those in *Brachypodium*, rice, and maize, which SBEI produces longer B-chains, whereas SBEII generates shorter A-chains [49,50]*.* In CS and *Ae. peregrina*, SBEII-a and SBEII-b may be responsible for the synthesis of longer B-chains, and SBEI may perform an important function in the synthesis of shorter A-chains. This hypothesis is supported by previous research in barley: Radchuk *et al*. [48] showed that SBEI expressed later than SBEIIs, and Regina *et al*. [29] suggested the reduction of SBEIIs led to a decrease of DP 10–18 chains in barley. Thus, the function of SBEs in wheat may be similar to that in barley, whereas the roles of SBEs in *Brachypodium* are the same as those in rice and maize. Although the sequences of SBEI and SBEIIs are different, the domains and 3D structure were similar, so it is possible that SBEs can have functions of mutual exchange in different species. DBEs are mutually complementary in the hydrolysis (debranching) of α-(1–6)-linkages in amylopectin and pullulan during formation of new chains (Figure 8) [51]. The details regarding the function of DBEs are not known. In this study, *ISA I*, *ISA II*, and *PUL* displayed a down-up expression pattern in Bd21, which may be responsible for the hydrolysis of starch in the pericarp, whereas they showed an up-down pattern in CS and *Ae. peregrina*. It is known that the starch content of the endosperm is less than 10% of the whole *Brachypodium* grain, much less than that in wheat (65–75%) [33]. On the other hand, there were only B-granules in *Brachypodium*, and the expression of starch synthesis-related genes was lower in the endosperm of *Brachypodium* compared to wheat and *Ae. peregrina*.

### Phosphorylation may play an important role in amylose synthesis

Protein phosphorylation, as the most common posttranslational modification in vivo, regulates and controls biological processes such as transcription and translation, cellular and communication, proliferation and differentiation [52]. Other studies proved that the enzymes (proteins) binding starch granules, such as SSI, SSII-a, SBEI, SBEII-a, and SBEII-b, can be phosphorylated and can participate in protein-protein interactions [53,54]. Grimaud *et al.* [55] showed that GBSSI can be stained with a phosphoprotein-specific dye in maize; however, phosphorylation sites in GBSSI have not been identified. In this study, we identified two GBSSI phosphorylation sites in CS and *Ae. peregrina*, including threonine 183 and tyrosine 185, and both of which are located within the starch synthase catalytic domain. Although no phosphorylated peptides were found at these positions in Bd21, sequence alignment suggests the Thr183 in CS and *Ae. peregrina* is replaced by Val in Bd21; this substitution may be responsible for the lack of phosphorylation sites in Bd21. Few studies have described how phosphorylation sites influence amylase activity. Some have indicated that the starch synthase catalytic domain is responsible for glucan-substrate recognition and affinity; meanwhile, Tetlow *et al.* [53] showed that phosphorylation improves amylase activity and increases amylose synthesis; moreover, recent studies of the interaction of the farnesyl moiety with the hydrophobic patch on 14-3-3 showed that phosphorylation increases affinity between the interacting proteins [56,57]. Finally, amylose content is lower in B-granules (~25%) than in A-granules (~30%) [58]. Thus, we hypothesize that the phosphorylation sites in the starch synthase catalytic domain may play an important role in recognizing and attracting glucan substrates. We also propose the exciting possibility that phosphorylation increases the activity of GBSSI in A-granules and thereby improves amylose synthesis there. The influence of different phosphorylation sites for amylase activity requires further study.

## Conclusions

We demonstrated the presence of only B-granules in Bd21, and they appear at ~8 DPA with a diameter of 4–6 μm. The expression of key genes in the studied genera is consistent with the dynamic development of starch granules. The expression of key genes in starch biosynthesis of Bd21 mainly occurs at early and intermediate stages, for the synthesis of starch in the pericarp and for the increase of the number or size of starch granules, respectively. In contrast, the high expression of biosynthetic genes at intermediate stages in CS and *Ae. peregrina* is mostly responsible for production of new B-granules and for the increase in the size of starch granules, respectively. The expression of the genes controlling synthesis of B1- or B2-chains occurs earlier compared to A-chains. GBSSI exists in B-granules of CS, in greater amounts compared to Bd21. There are two phosphorylation sites (Thr183 and Tyr185) in *Triticum* and *Aegilops*, whereas Thr183 was replaced by Val in Bd21. Phosphorylation of GBSSI may play a central role in amylose synthesis.

## Methods

### Plant materials, planting, and sampling

A diploid *B. distachyon* inbred line Bd21, the common wheat variety (*Triticum aestivum* L., 2n = 6× = 42, AABBDD) Chinese Spring (CS), and *Aegilops peregrina*, accession # AP200201 (2n = 4× = 28, SSUU) were used in this study. *Ae. peregrina* was kindly provided by the Department of Plant Breeding, Technical University of Munich, Germany. The grains were first stratified at 4°C for 7 d on moist paper to promote synchronous germination, then transferred to soil and grown in a growth chamber at 21/18°C (day/night) and 65% relative humidity under a short-day (8/16 h light/dark) photoperiod with light intensity of 120 μmol m^−2^∙s^−1^ for 4 weeks. The plants were then switched to long-day conditions with a 16/8 h light/dark photoperiod and the same light intensity. The plants were irrigated twice a week with a mineral nutrient solution. To harvest grains at defined developmental stages, individual flowers were tagged using colored tape at various stages post-anthesis. Grains were harvested at 4, 6, 8, 10, 12, 14, 16, 18, 20, 22, 25, and 30 days post-anthesis (DPA).

### Light microscopy and scanning electron microscopy (SEM)

A modification of Guillon *et al*. [33] was used for conventional chemical fixation. Grains were cut in transverse slices, approximately 3–4 mm thick, fixed in 3% (w/v) glutaraldehyde and 4% paraformaldehyde in 0.1 M PBS (pH 7.4) overnight, after which samples were directly dehydrated in a series of ethanol solutions in water and then infiltrated and polymerized in medium-grade LR White resin. For light microscopy, sections approximately 1-μm thick were prepared, and 10% (w/v) fast green and I_2_ were used to stain the proteins and the starchy endosperm. In this study, three grains, nine sections, and ~600 starch granules were used to quantify starch granule sizes at each stage.

Starch granules from different species and various grain developmental stages were dusted on the surface of a carbon-adhesive tab and sputter-coated with gold-palladium particles using Dentum Vacuum Desk II. SEM examination of starch granules was performed using a Hitachi Model S-4700 scanning electron microscope at 10.0 kV.

### Purification of starch granules

Starch granules were separated and purified according to Branlard *et al*. [59] and Bancel *et al.* [60] with some modifications. The seeds were manually crushed and then soaked overnight in 1 mL of water at 4°C. After centrifugation, 500 μL of water was added to the pellet. The slurry was layered on 1 mL of 80% (w/v) CsCl and centrifuged at 3500 × *g* for 5 min. The precipitate containing the starch granules was then washed 3 times with 1 mL of washing buffer (55 mM Tris–HCl pH 6.8, 2.3% [w/v] SDS, 1% [w/v] dithiothreitol [DTT], 10% [v/v] glycerol) for 30 min at 20°C. At the beginning of each washing step, the granules were disrupted using sonication by means of an ultrasonic processor (Vibracell, VC50, Bioblock Scientific, Illkirch, France) at the power of 20 W with a 20-s pulse before continuous mixing. The granules were washed three times (10 min each time), and then washed three times (5 min each time) with cold water, once with cold acetone, and finally were air-dried. Each washing step was followed by centrifugation at 3500 × *g* for 5 min. All washing and centrifugation steps were carried out at room temperature to avoid precipitation of SDS. The purity of the starch fraction was monitored using SEM.

### Chromosomal locations, analysis of motifs, and construction of a phylogenetic tree

The locations of the motifs in starch synthesis-related genes were confirmed using the database containing complete *B. distachyon* genome sequences [31]. Using the ClustalW software, we performed multiple alignments of the identified *B. distachyon* amino acid sequences of the genes related to starch synthesis with those from wheat and rice [61] using default options in the software. The phylogenetic tree was constructed using the bootstrap neighbor-joining (NJ) method with a Kimura 2-parameter model in the MEGA 4.0 software [62].

### RNA extraction, cDNA synthesis, and quantitative real-time PCR (qRT-PCR)

Developing grains from the central part of the spikes in Bd21, CS, and *Ae. peregrina* were harvested at 12 developmental stages of the grain (4, 6, 8, 10, 12, 14, 16, 18, 20, 22, 25, and 30 DPA). Total RNA of individual samples from different grain-developmental stages were extracted with TRIzol Reagent according to the manufacturer’s instructions (Invitrogen), and the purification of mRNA was performed, as described by Li *et al.* [63]. With oligo(dT) and random primers, the mRNA was used to synthesize cDNA from approximately 100 ng mRNA using the Superscript first-strand synthesis kit (Promega, Madison, WI, USA). The resulting cDNA was used for qRT-PCR analysis.

Transcriptional expression patterns of these genes were detected using qRT-PCR according to Paolacci *et al.* [64] with minor modifications, according to a 4-step protocol with a melting curve analysis: (1) initial incubation at 94°C for 3 min, 40 cycles of (2) denaturation at 94°C for 15 s, (3) hybridization at 58°C for 15 s, and (4) extension at 72°C for 20 s. The fluorescence signal was acquired at the end of the extension in every cycle. Specific primer pairs were designed for the genes using the Primer5.0 software by imposing the following stringent criteria: melting temperature of 58 ± 2°C, PCR amplicon length between 80 and 300 bp, primer length of 22 ± 4 bases, and 40–60% guanine-cytosine content. Primers were also designed within the 3′ region of each sequence to encompass all potential splice variants and to ensure equal RT efficiency. The complete set of primer pairs used for qRT-PCR analysis is shown in Additional files 8 and 9. The specific primers had a unique melting temperature peak. The efficiency of the primers was determined by means of standard curves using serial α-gliadin gene dilutions; the efficiency ranged from 90% to 110% (Additional file 8 and 9), and R2 values (coefficient of determination) were calculated for standard curves higher than 0.993 for expression analysis of the genes and the candidate reference genes (Additional file 8 and 9). Triplicate PCRs were performed for each gene.

### Protein extraction and identification using tandem mass spectrometry (MS/MS)

The seeds were manually crushed and soaked overnight in 1 mL of washing buffer (70 mmol Tris–HCl pH 6.8, 2% SDS, and 2% β-mercaptoethanol) at 4°C. After centrifugation at 16000 rpm for 1 min, 1 mL of washing buffer was added to the pellets, and the mixture was vibrated. After centrifugation (16000 rcf for 1 min) and air drying, 1 mL of the extraction solution (70 mmol Tris–HCl pH 6.8, 2% SDS, 2% β-mercaptoethanol, 20% glycerin, 0.005% bromophenol blue) was added, and then the pellets were boiled at 100°C for 4 min. After centrifugation at 16000 rpm for 1 min, the supernatant was stored at 4°C.

Protein bands were manually excised from the gels, and after trypsin digestion, these were analyzed on a 4800 Plus MALDI TOF/TOF Analyzer (Applied Biosystems, USA). All MS spectra were used for search in the NCBI database Viridiplantae (900091) and *Triticum* (16682) via the MASCOT software with GPS Explorer software version 2.0 (Applied Biosystems). The peptide tolerance was set as 100 ppm and fragment mass tolerance was 0.4 Da. One missed cleavage was allowed, and carbamidomethyl (Cys) and oxidation (Met) were specified as variable modifications. MASCOT scores greater than 65 (*p* < 0.05) were accepted [65]. Protein identification by MALDI-TOF–MS is shown in Additional file 6.

### Antibody development, Western blot, and immunolabeling

The monoclonal antibody against GBSSI (peptide SEWDPAKDKFLA) was developed by Abmart (Shanghai, CHINA). The extracted proteins described above were separated by SDS-PAGE. After silver staining, the expected protein bands were collected and digested by trypsin, and then identified by MALDI-TOF/TOF-MS as described above. The identified GBSSI was further confirmed by western blotting according to Li *et al*. [63].

Cross-sections of 12-day-old immature grains were immediately cut into small pieces (~1 mm^3^) and fixed with 4% glutaraldehyde in 0.1 M PBS (pH 7.4) for 12 h. Then, the samples were directly dehydrated in a series ethanol solutions, and permeated and polymerized in medium-grade LR White resin. Ultrathin (70 nm) sections were prepared on an ultramicrotome (Leica EM UC6) with a diamond knife and placed on Formvar-coated grids. Subsequently, the grids were rinsed for 2 × 5 min in PBG (0.1 M PBS with 35 mM glycine), and transferred to PBT (0.1 M PBS, 0.5% BSA, and 0.02% Tween 20) 2 × 5 min, then incubated overnight with an anti-GBSSI antibody diluted 1:1000. The sections were rinsed 6 × 5 min in PBT the next day, and the grids were incubated with a secondary antibody: a goat anti-mouse IgG antibody conjugated to colloid gold (Auroprobe-EM, GAR-G15; Janssen, Belgium) diluted 1:20 in the blocking solution (5% skimmed milk, 0.1% Tween 20, and 0.2 M PBS) for 15 min. The sections were washed 2 × 2 min in PBT and 2 × 2 min in PBG followed by glass-distilled water, and then poststained with 2% uranyl acetate for 10 min. Finally, the grids with sections were washed 6 × 5 min in PBG and were allowed to air dry before examination under the scanning transmission electron microscope. In the control experiment, the primary antibody was omitted to test for nonspecific secondary antibody binding [66,67].

### Identification of a phosphorylated peptide using liquid chromatography (LC) with MS/MS

The identified GBSSI protein bands identified on the SDS-PAGE gel were cut into small pieces (~3–4 mm^3^), then washed 2 × 10 min in water. Then the minced gel was washed for 10 min in 200 μL of 50 mM NH_4_HCO_3_:ACN (1:1), and we repeated this step until all the color was gone. We then added 10 mM DTT (10 μL 1 M DTT) and 990 μL 50 mM NH_4_HCO_3_ to the tube and kept the gels at 56°C for 1 h. After cooling to room temperature, we removed the supernatant, added 50 μL Iodoacetamide (IAM) (22 μL 1 M IAM, 378 μL 50 mM NH_4_HCO_3_), and incubated the mixture in the dark for 45 min. After that, we used 25 mM NH_4_HCO_3_, 50% ACN, and ACN in this order to wash the strips, and vacuum-dried them for 5 min. Finally, trypsin hydrolysis was conducted at 4°C for 60 min and at 37°C for 16–18 h in this order.

For MS analyses, peptides were resuspended in 0.1% formic acid (FA) and analyzed on an LTQ Orbitrap Elite mass spectrometer (Thermo Fisher Scientific) coupled online to an Easy-nLC 1000 (Thermo Fisher Scientific) in the data-dependent mode. The sample was trapped on a 150-μm × 0.5-mm precolumn and eluted from an analytical 75-μm × 150-mm column. Peptides were separated using a linear gradient formed by 2% ACN (acetonitrile) in 0.1% FA (mobile phase A) and 98% ACN in 0.1% FA (mobile phase B), from 3% to 30% of mobile phase B in 90 min. The mass spectrometer was operated with full scan acquisition in the Orbitrap at 24000 Da resolution (350–1800 *m/z*). The mass spectrometer was set up to acquire collision-induced dissociation (CID) MS/MS scans after each MS1 scan of the 25 most abundant ions with MSA central losses of m/z 98, 49, and 32.6. For CID, the normalized collision energy was set to 35.

The MS data were analyzed with MaxQuant software, version 1.3.0.5, and were used to search the NCBI database. During the searching, the enzyme specificity was set to trypsin with the maximum number of missed cleavages of 2. Oxidized methionines, phosphorylation addition to serine, threonine, or tyrosine, and N-terminal protein acetylation were included in the search as variable modifications. Carbamidomethylation of cysteines was included in the search as a fixed modification. The false discovery rate (FDR) for peptides, proteins, and site identification was set to 1%.

## Additional files

Additional file 1:
**Development of seeds.** (A) Whole seeds at the 13 stages of seed development in *Brachypodium distachyon* Bd21, Chinese Spring (common wheat), and *Aegilops peregrina*. (B) Changes in fresh weight of developing seeds. Error bars represent SD of 3 replicates. Additional file 2:
**Micrographs of peripheral cell layers at the early stages of grain development in** ***Brachypodium distachyon*** **Bd21, Chinese Spring (CS; common wheat), and** ***Aegilops peregrina.*** Legend: DPA, days post-anthesis; en, endosperm; nu, nucellus tissue; pe, pericarp; ne, nucellar epidermis. Additional file 3:
**Chromosomal locations of the key genes in starch biosynthesis annotated along the 5 chromosomes of** ***Brachypodium distachyon*** **Bd21.** Chromosome numbers and sizes (Mb) are indicated at the bottom of each bar. Additional file 4:
**Analysis of motifs of key genes in** ***Brachypodium distachyon*** **Bd21.**
Additional file 5:
**Phylogenetic analysis of key genes in starch biosynthesis among** ***Brachypodium,*** **wheat, rice and maize.** a. Phylogenetic tree was constructed based on the nucleotide sequences of 70 key genes in starch biosynthesis from *Brachypodium*, wheat, rice and maize. b–g. *GBSSI*, *SSI*, *SBEI*, *SBEII-a*, *ISAI*, *PUL*, and *AGPL* were selected to construct different phylogenetic trees. Additional file 6:
**Protein identification by matrix-assisted laser desorption ionization time-of-flight mass spectrometry (MALDI-TOF MS).**
Additional file 7:
**Phosphorylation identification of granule-bound starch synthase (GBSSI) in Chinese Spring (common wheat) and in** ***Aegilops peregrina.*** Lowercase t and y represent the phosphorylation sites. Additional file 8:
**Efficiency and R2 values (coefficient of determination) of primer pairs.** Data were calculated from standard curves (5-fold dilution series from pooled cDNAs) in *Brachypodium distachyon* Bd21. Additional file 9:
**Efficiency and R2 values (coefficient of determination) of primer pairs, when calculated for standard curves (5-fold dilution series from pooled cDNAs) in Chinese Spring (common wheat) and** ***Aegilops peregrina.***
