# Supplementary figures and images for: Dynamic development of starch granules and the regulation of starch biosynthesis in Brachypodium distachyon: comparison with common wheat and Aegilops peregrina
Source: BMC Plant Biol. 2014 Aug 6;14:198. doi: 10.1186/s12870-014-0198-2 (PMC4256708; doi:10.1186/s12870-014-0198-2)

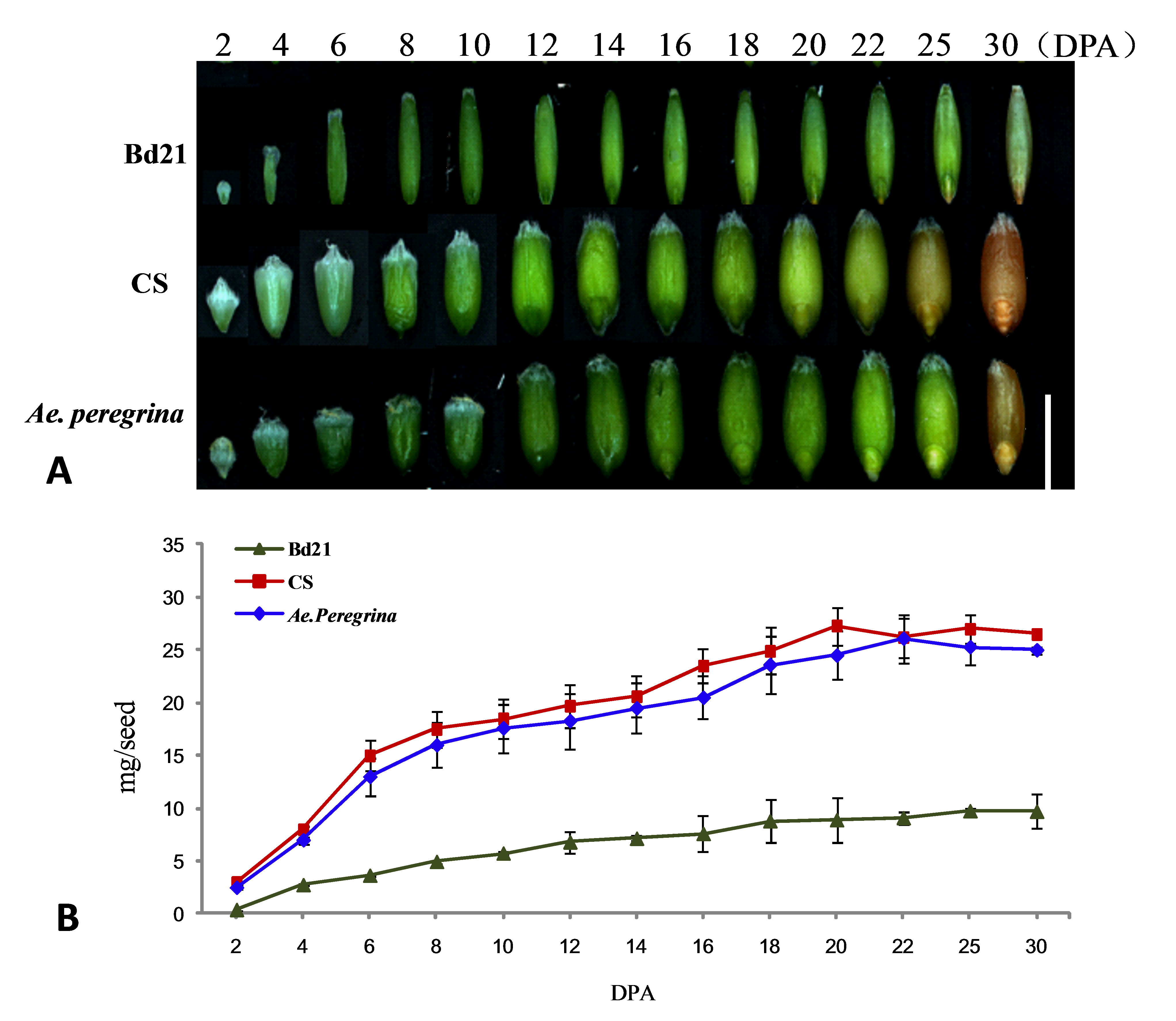

Supplement: Additional file 1: — Development of seeds. (A) Whole seeds at the 13 stages of seed development in Brachypodium distachyon Bd21, Chinese Spring (common wheat), and Aegilops peregrina. (B) Changes in fresh weight of developing seeds. Error bars represent SD of 3 replicates. [file 12870_2014_198_MOESM1_ESM.jpeg]

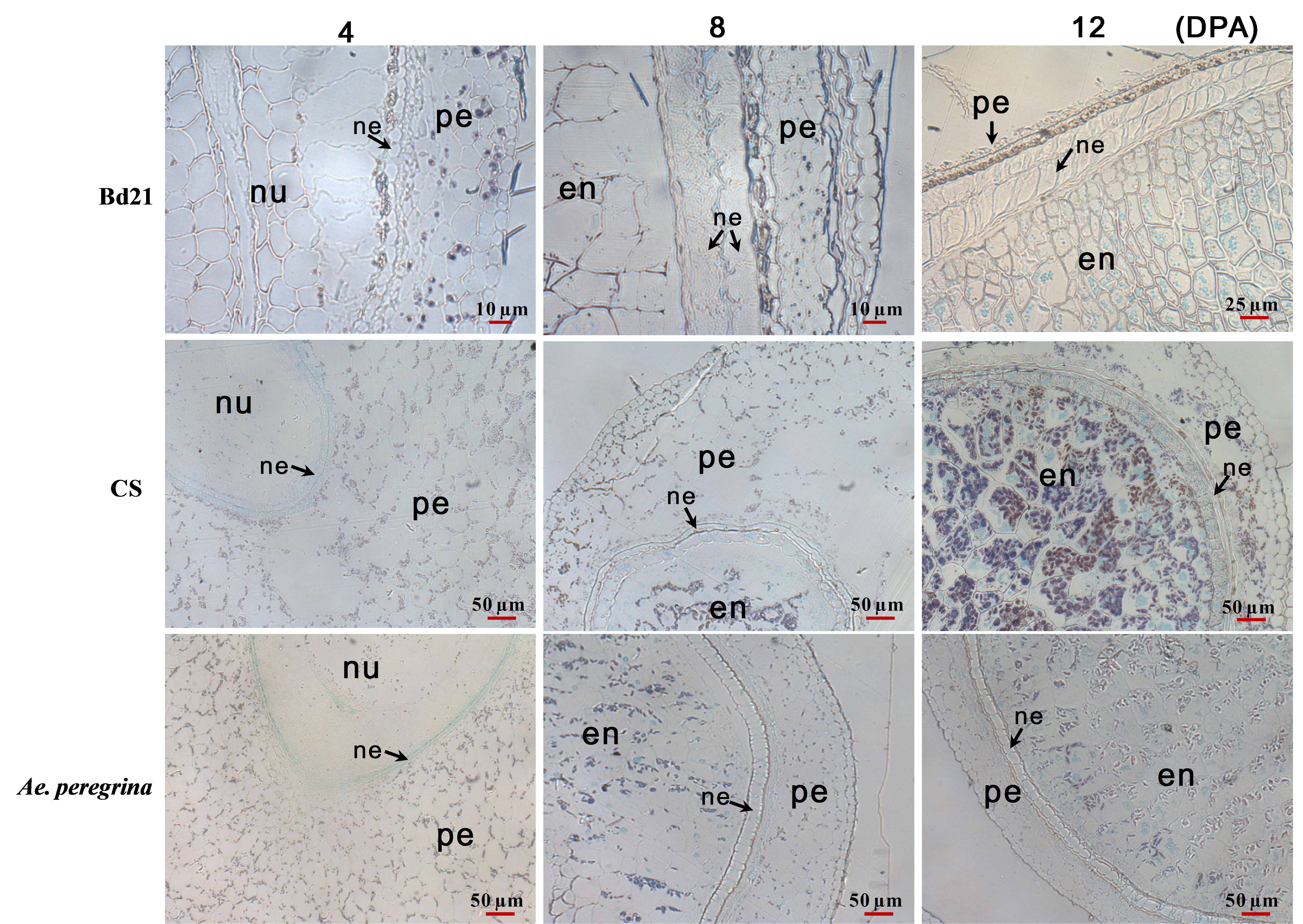

Supplement: Additional file 2: — Micrographs of peripheral cell layers at the early stages of grain development in Brachypodium distachyon Bd21, Chinese Spring (CS; common wheat), and Aegilops peregrina. Legend: DPA, days post-anthesis; en, endosperm; nu, nucellus tissue; pe, pericarp; ne, nucellar epidermis. [file 12870_2014_198_MOESM2_ESM.jpeg]

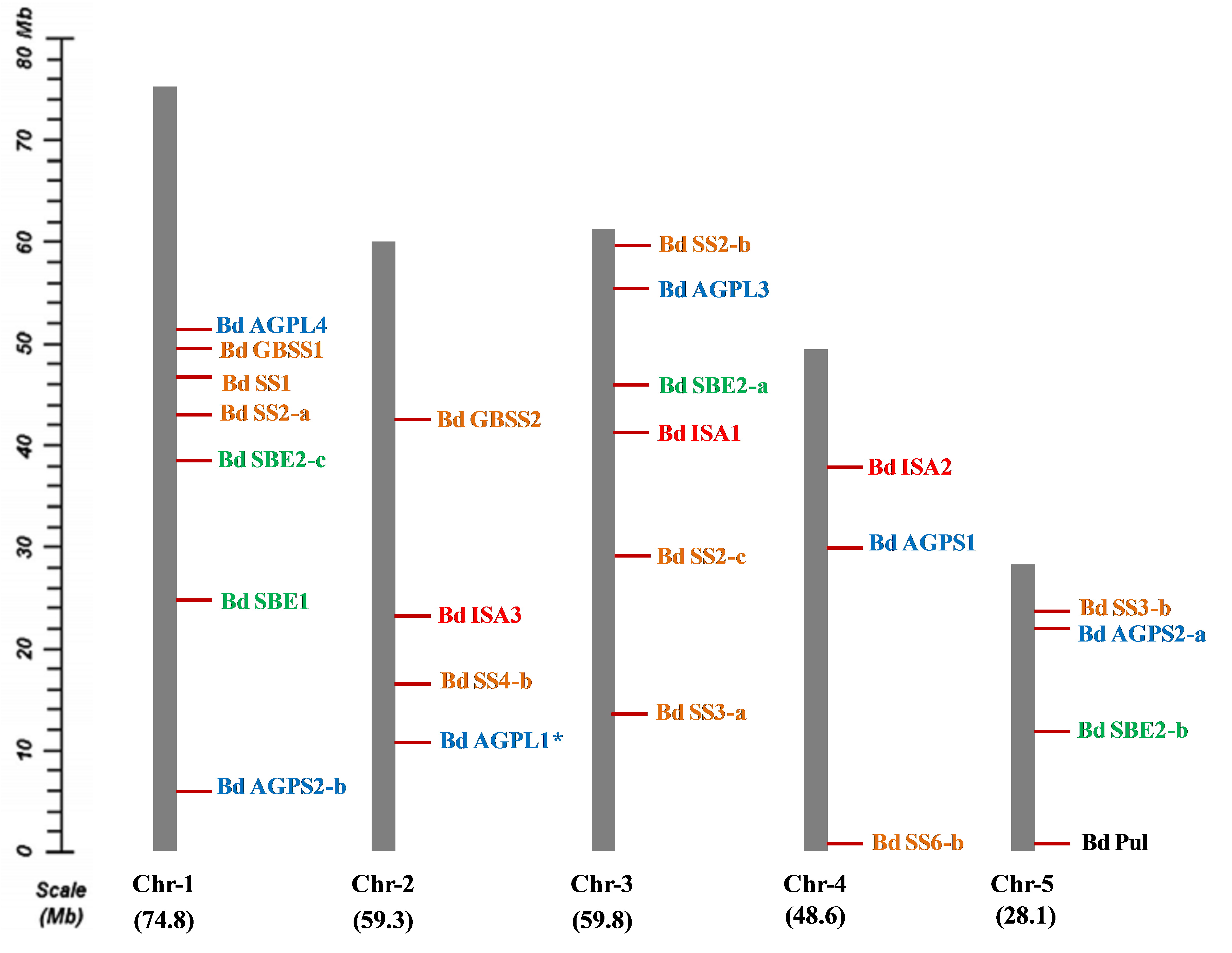

Supplement: Additional file 3: — Chromosomal locations of the key genes in starch biosynthesis annotated along the 5 chromosomes of Brachypodium distachyon Bd21. Chromosome numbers and sizes (Mb) are indicated at the bottom of each bar. [file 12870_2014_198_MOESM3_ESM.jpeg]

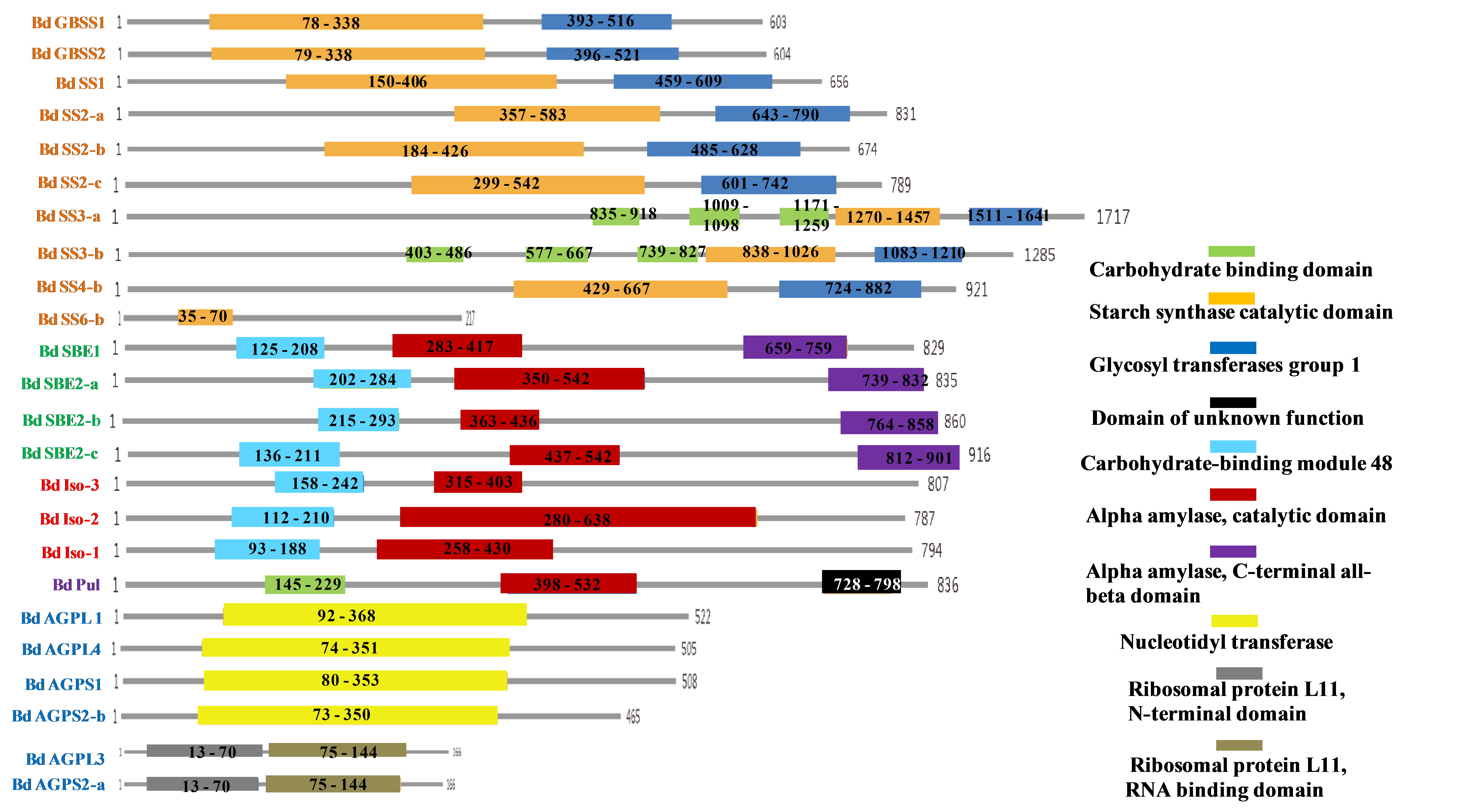

Supplement: Additional file 4: — Analysis of motifs of key genes in Brachypodium distachyon Bd21. [file 12870_2014_198_MOESM4_ESM.jpeg]

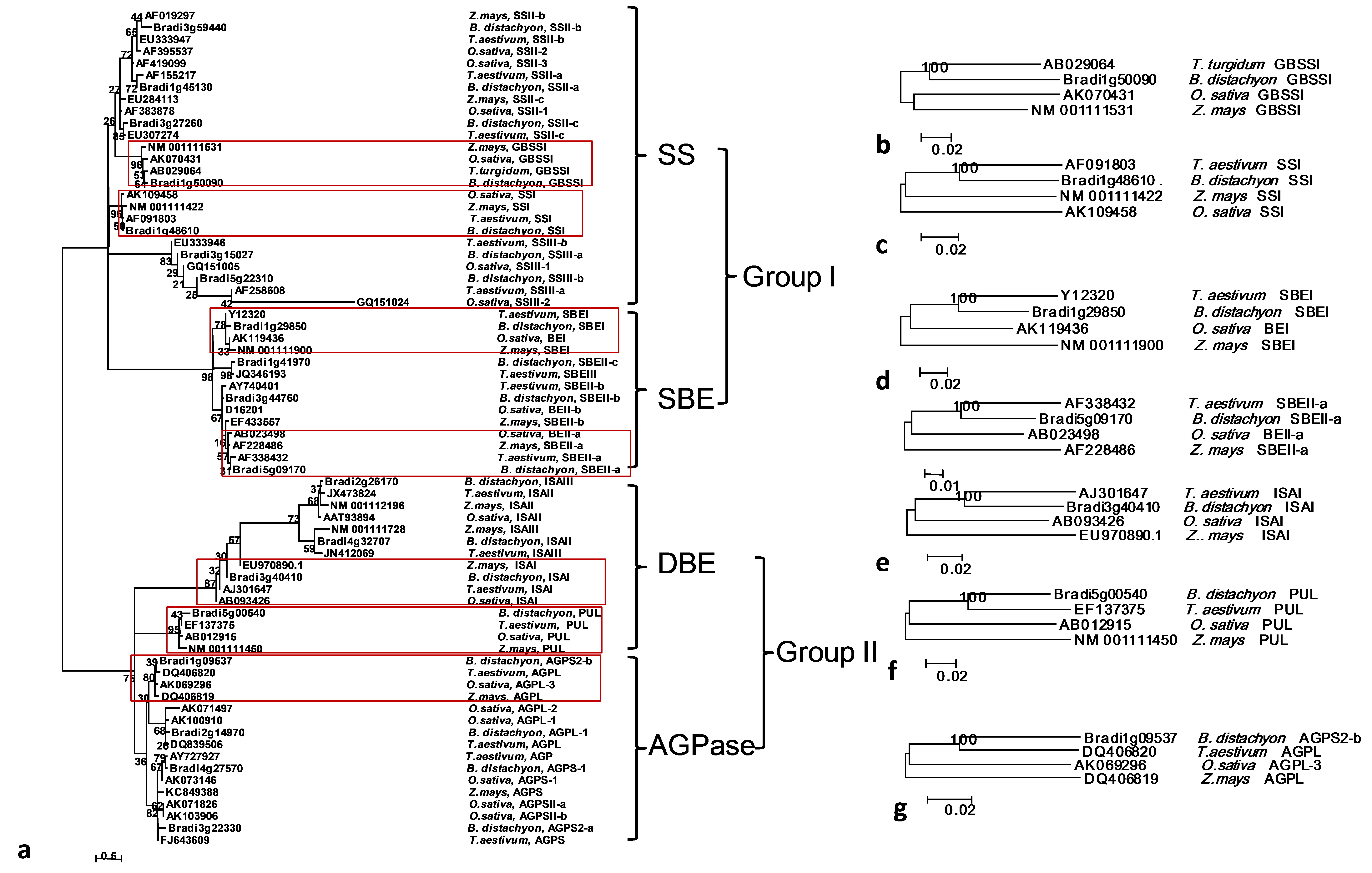

Supplement: Additional file 5: — Phylogenetic analysis of key genes in starch biosynthesis among Brachypodium, wheat, rice and maize. a. Phylogenetic tree was constructed based on the nucleotide sequences of 70 key genes in starch biosynthesis from Brachypodium, wheat, rice and maize. b–g. GBSSI, SSI, SBEI, SBEII-a, ISAI, PUL, and AGPL were selected to construct different phylogenetic trees. [file 12870_2014_198_MOESM5_ESM.jpeg]
