# Supplementary material for: Dynamic development of starch granules and the regulation of starch biosynthesis in Brachypodium distachyon: comparison with common wheat and Aegilops peregrina
Source: BMC Plant Biol. 2014 Aug 6;14:198. doi: 10.1186/s12870-014-0198-2 (PMC4256708; doi:10.1186/s12870-014-0198-2)
Supplement: Additional file 6: — Protein identification by matrix-assisted laser desorption ionization time-of-flight mass spectrometry (MALDI-TOF MS). [file 12870_2014_198_MOESM6_ESM.pdf]

Table 1. Protein identification from SDS-PAGE bands by MALDI-TOF-MS.

Spot no.	Accession no.	Protein name	Species	Protein score[a]	Protein score C.I.%	Total Ion score	Total ion score CI%	Number of matching peptides	Sequence coverage %	Protein PI	Protein MW (kDa)
1	gi|298543883	starch synthase II	*Triticum aestivum*	143	100	106	100	10	12.5	6.27	87.139
2	gi|58618129	starch branching enzyme IIb	*Triticum aestivum*	166	100	141	100	9	11.1	5.75	94.629
3	gi|399153332	waxy protein	*Triticum aestivum*	649	100	468	100	22	36.7	7.85	67.165
4	gi|452113211	waxy protein	*Aegilops*	518	100	401	100	16	26.5	7.85	67.100
5	gi|357110669	granule-bound starch synthase 1	*Brachypodium distachyon*	128	100	86	99.9	15	25	7.12	66.219

a: Protein Score: statistical probability of true positive identification of the predicted protein calculated by MASCOT (score ≥ 42 against NCBInr).
