# Supplementary material for: Dynamic development of starch granules and the regulation of starch biosynthesis in Brachypodium distachyon: comparison with common wheat and Aegilops peregrina
Source: BMC Plant Biol. 2014 Aug 6;14:198. doi: 10.1186/s12870-014-0198-2 (PMC4256708; doi:10.1186/s12870-014-0198-2)
Supplement: Additional file 7: — Phosphorylation identification of granule-bound starch synthase (GBSSI) in Chinese Spring (common wheat) and in Aegilops peregrina. Lowercase t and y represent the phosphorylation sites. [file 12870_2014_198_MOESM7_ESM.pdf]

species	Sequence	pRS Probability	pRS Site Probabilities	pRS Score	MH+ [Da]	RT [min]
CS	IYGPDAG**t**DYEDNQQR	1.0	97.0	159.406	1921.75	38.79
CS	IYGPDAGTD**y**EDNQQR	0.90	89.70	29.86	1921.75	39.09
Ae. peregrina	EKIYGPDAG**t**DYEDNQQR	1.0	99.8	71.93	2178.89	35.21
Ae. peregrina	IYGPDAGTD**y**EDNQQR	1.0	97.1	121.39	1921.75	38.75
