# Supplementary material for: Dynamic development of starch granules and the regulation of starch biosynthesis in Brachypodium distachyon: comparison with common wheat and Aegilops peregrina
Source: BMC Plant Biol. 2014 Aug 6;14:198. doi: 10.1186/s12870-014-0198-2 (PMC4256708; doi:10.1186/s12870-014-0198-2)
Supplement: Additional file 8: — Efficiency and R2 values (coefficient of determination) of primer pairs. Data were calculated from standard curves (5-fold dilution series from pooled cDNAs) in Brachypodium distachyon Bd21. [file 12870_2014_198_MOESM8_ESM.pdf]

Gene	Forward primer(5'-3')	Reverse primer(5'-3')	Amplicon length (bp)	Efficiencies	R_2
Ubi4	TGACACCATCGACAACGTGA	GAGGGTGGACTCCTTCTGGA	126 bp	99.4%	0.996
GAPDH	TTGCTCTCCAGAGCGATGAC	CTCCACGACATAATCGGCAC	236bp	94.3%	0.997
GBSS Ⅰ	CGACAACAACCCGTTCTACTCAG	ATCCCGCCCTTCATCCAG	300bp	97.4%	1.000
SS Ⅰ	CCTTGTGCCAGTCCTTCTT	GCTCTACACCCTGATGTGCTAA	104bp	94.7%	0.998
SSⅡa	CCGCTCTGTTTTGGTGAT	TCGTTCTGGCGTATGATG	255bp	103.4%	0.995
SSⅡb	GACCGAGTGGTGACCGTGAG	GTTCTGGTTTATGATGTCGTGGAG	90bp	97.3%	0.994
SSⅡc	GATGGTGTCCGTGTTTCC	AGGTCATAGCCAATCTCGTT	221bp	105.2%	0.992
SSⅢa	GCAGGCATACAGATTAGAC	CTGGCTGAACCCAACACA	306bp	97.6%	0.992
SBE1	CGAGTGGTCAATGAGTGG	CGAAGCAGGCTGTAAGTC	172bp	92.4%	0.998
SBE2a	TGACCCAACTCTGAAAGC	TGAAGTCGCCTACTAACG	209bp	99.9%	0.995
SBE2b	AATCACCGACAGCGTAAC	CATCCAAACCACCTTCAT	200bp	102.6%	0.999
SBE3	GGTCCGCAGACAGAAGAG	TCAACCCAGTAGGCAAGG	215bp	102.7%	0.995
ISA1	TACTTCACCTCCTCTTCG	CGCCCACAGTTGTTTATC	104bp	92.1%	0.998
ISA2	ATGACCGAGGTATGTGGC	GAGGGTGACGGAAGAAGC	150bp	105.2%	0.985
ISA3	TCGTGCTCTGCCTGTATG	AATGATCCGAGGAAACTCA	268bp	108.1%	0.989
PUL	GCAGTTACAAAGCGTTCA	TGTCATCCAATACACCAG	141bp	95.3%	0.992
