# Supplementary material for: Dynamic development of starch granules and the regulation of starch biosynthesis in Brachypodium distachyon: comparison with common wheat and Aegilops peregrina
Source: BMC Plant Biol. 2014 Aug 6;14:198. doi: 10.1186/s12870-014-0198-2 (PMC4256708; doi:10.1186/s12870-014-0198-2)
Supplement: Additional file 9: — Efficiency and R2 values (coefficient of determination) of primer pairs, when calculated for standard curves (5-fold dilution series from pooled cDNAs) in Chinese Spring (common wheat) and Aegilops peregrina. [file 12870_2014_198_MOESM9_ESM.pdf]

Gene	Forward primer(5'-3')	Reverse primer(5'-3')	Amplicon length (bp)	Efficiencies （CS）	R_2 （CS）	Efficiencies （Ae.P）	R_2 （Ae.P）
ADP	GCTCTCCAACAACATTGCCAAC	GCTTCTGCCTGTCACATACGC	126 bp	100.7%	0.999	100.9%	0.991
Ubi4	TGACACCATCGACAACGTGA	GAGGGTGGACTCCTTCTGGA	126 bp	96.7%	0.983	98.9%	0.990
GBSS Ⅰ	CTGGTCACGTCCCAGCTC	ACGAACACGAGGTTCATGC	321bp	90.2%	0.999	105.2%	0.998
SS Ⅰ	GCAAAAGGAGAGGAGGGTACA	ACGTATGGTCTTTCGTCATGC	236bp	92.4%	0.995	99.5%	0.996
SS Ⅱ a	CCTGAGCACTACCTGGAACACTT	TCTGCCGTATGATGTCGTGAA	175bp	92.0%	0.992	102%	0.991
SS Ⅱ b	GCTGAAGACGGTGGAAGGC	TCGGGTGTAGTTGGTGTAGTCG	154bp	100.4%	0.992	101.6%	0.989
SS Ⅱ c	ACACTGCTGCTAAACGCTAT	TTGGCTCTGACAGGAAAGAT	209bp	99.9%	0.994	103.0%	0.986
SSⅢa	AGTCGGAGGGTTGGTTTA	ATCCCATTCCTGTTGTCATA	196bp	97.4%	0.996	97.1%	0.983
SBE1	GGCCCAGTACAACGATCACT	CTTTGACACGAGTGGCTTCA	257bp	105.3%	0.997	100.3%	0.990
SBE2a	TCGTGGCATAGCATTACAT	GAGTTTGCGGACCTCTTG	239bp	93.3%	0.997	102.8%	0.989
SBE2b	CTTTGGTGGATTTGGTAGG	TTAGTTCATTGGAGCATAGACA	130bp	91.5%	0.994	99.7%	0.989
SBE3	CAGTTCATCGCCTTCCTA	CCTCATTTCAACCCAGTAA	133bp	102.1%	0.983	102.3%	0.999
ISA1	TGAATCGGACTGGGAACG	CACCACGACATTGGAAACAT	131bp	94.5%	0.998	96.5%	0.996
ISA2	AGAGGCTAAGGCAGATAAGG	CGAACCAGCAGAGGAGTG	291bp	100.2%	0.997	101.2%	0.997
ISA3	TGCGGGAATACACTAAACT	GAGGAGCATCAAGAGGACTA	157bp	106.9%	0.985	103.6%	0.998
PUL	TCATCTCCGCTTTTTCGTCT	TACTTCGTGCGGACACACAT	272bp	96.5%	0.997	98.7%	0.997
